# Deep learning-based image enhancement for improved black blood imaging in brain metastasis

**DOI:** 10.1007/s00330-025-11920-7

**Published:** 2025-08-08

**Authors:** Gaeun Oh, Seungyoon Paik, Sang Won Jo, Hye Jeong Choi, Roh-Eul Yoo, Seung Hong Choi

**Affiliations:** 1 Seoul National University College of Medicine, Seoul, Republic of Korea; 2 Department of Radiology, Dongtan Sacred Heart Hospital, Hallym University College of Medicine, Hwaseong-si, Gyeonggi-do Republic of Korea; 3 Department of Radiology, CHA University Bundang Medical Center, Seongnam-si, Republic of Korea; 4 Department of Radiology, Seoul National University Hospital, Seoul, Republic of Korea; 5 Center for Nanoparticle Research, Institute for Basic Science (IBS), Seoul, Republic of Korea; 6 School of Chemical and Biological Engineering, Seoul National University, Seoul, Republic of Korea

**Keywords:** Brain neoplasm, Magnetic resonance imaging, Deep learning, Image enhancement

## Abstract

**Objectives:**

To evaluate the utility of a deep learning (DL)-based image enhancement for improving the image quality and diagnostic performance of 3D contrast-enhanced T1-weighted black blood (BB) MR imaging for brain metastases.

**Materials and methods:**

This retrospective study included 126 patients with and 121 patients without brain metastasis who underwent 3-T MRI examinations. Commercially available DL-based MR image enhancement software was utilized for image post-processing. The signal-to-noise ratio (SNR) and contrast-to-noise ratio (CNR) of enhancing lesions were measured. For qualitative assessment and diagnostic performance evaluation, two radiologists graded the overall image quality, noise, and artifacts of each image and the conspicuity of visible lesions. The Wilcoxon signed-rank test and regression analyses with generalized estimating equations (GEEs) were used for statistical analysis.

**Results:**

For MR images that were not previously processed using other DL-based methods, SNR and CNR were higher in the DL-enhanced images than in the standard images (438.3 vs. 661.1, *p* < 0.01; 173.9 vs. 223.5, *p* < 0.01). Overall image quality and noise were improved in the DL images (*p* < 0.01, average score-5 proportion 38% vs. 65%; *p* < 0.01, 43% vs. 74%), whereas artifacts did not significantly differ (*p* ≥ 0.07). Sensitivity was increased after post-processing from 79 to 86% (*p* = 0.02), especially for lesions smaller than 5 mm (69 to 78%, *p* = 0.03), and changes in specificity (*p* = 0.24) and average false-positive (FP) count (*p* = 0.18) were not significant.

**Conclusion:**

DL image enhancement improves the image quality and diagnostic performance of 3D contrast-enhanced T1-weighted BB MR imaging for the detection of small brain metastases.

**Key Points:**

***Question*** *Can deep learning (DL)-based image enhancement improve the image quality and diagnostic performance of 3D contrast-enhanced T1-weighted black blood (BB) MR imaging for brain metastases?*

***Findings*** *DL-based image enhancement improved image quality of thin slice BB MR images and sensitivity for brain metastasis, particularly for lesions smaller than 5* *mm*.

***Clinical relevance*** *DL-based image enhancement on BB images may assist in the accurate diagnosis of brain metastasis by achieving better sensitivity while maintaining comparable specificity*.

**Graphical Abstract:**

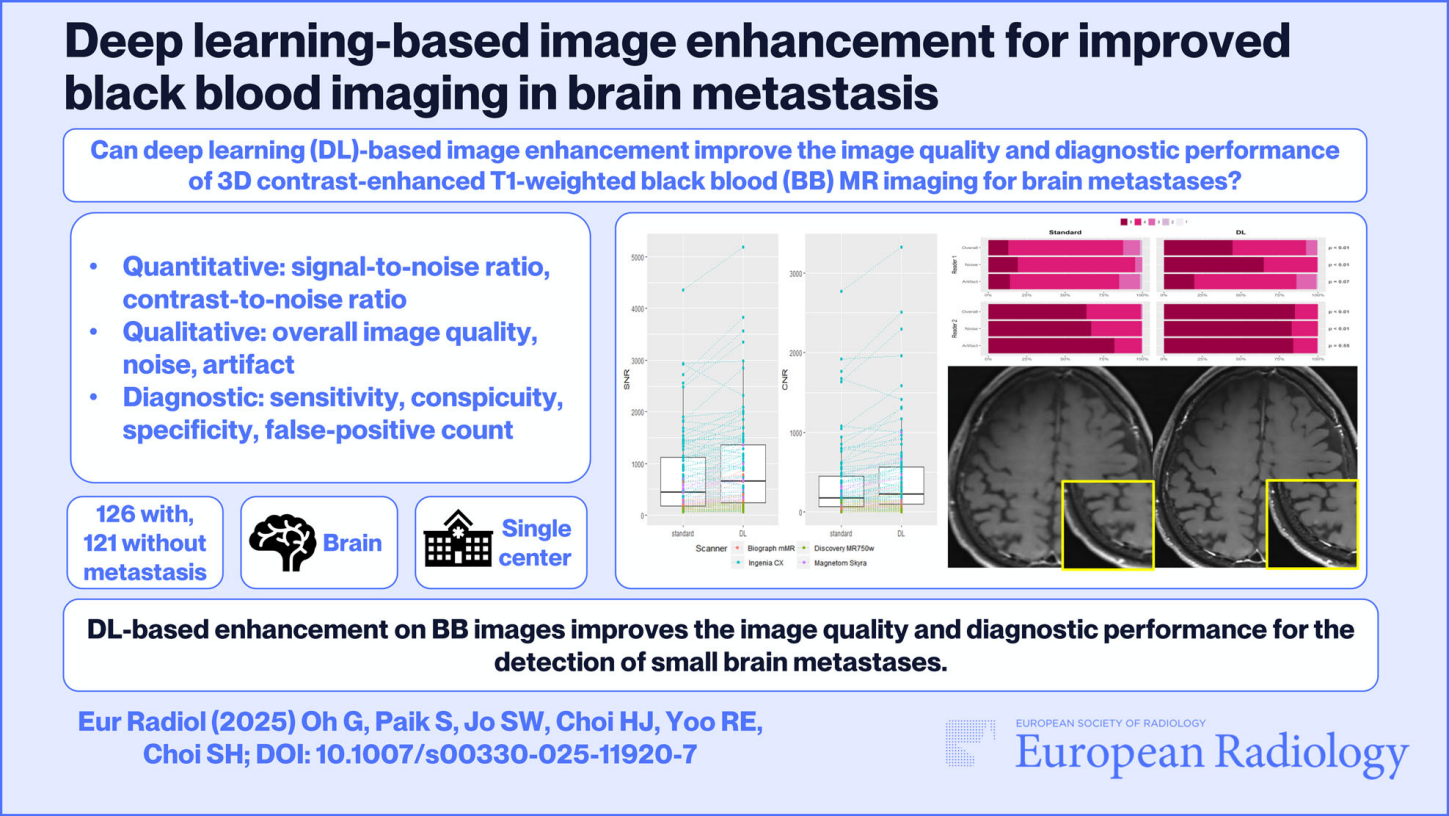

## Introduction

Metastatic brain tumors are among the most common intracranial tumors in adults. Although approximately 20% of cancer patients are known to develop brain metastases, its percentage is likely to be underreported and may increase as primary cancer survival rates improve [1, 2]. Today, treatments are individualized, with options including chemotherapy, whole-brain radiation therapy, stereotactic radiosurgery, and surgical resection. Accurate information regarding the location, size, and number of brain metastases is essential for determining the most appropriate treatment [3, 4].

Black blood (BB) MR imaging is a useful technique for visualizing brain metastases. Common methods of BB imaging involve variable refocusing of flip-angles for effective intravoxel dephasing or preparation pulses, such as in double inversion recovery or motion-sensitized driven equilibrium [5]. These methods mostly make use of the blood flow property. In typical contrast-enhanced T1-weighted brain MR images, both vessels and metastases show high signal intensities. However, vessel signals are almost exclusively suppressed in BB images, allowing improved contrast between brain vessels and metastatic lesions. Previous studies have shown that metastases are better detected on BB images [6–9] than on conventional magnetization-prepared rapid gradient echo MR images.

Thin-slice MR images have shown superior diagnostic performance in detecting small lesions by reducing the partial volume artifact [10, 11]. In particular, previous studies on brain metastases have shown that using thicker slices may increase the chances of leaving out small metastases, inappropriately determining the extent of each lesion, or mistaking vessels for metastases [12, 13]. Hence, the Response Assessment in Neuro-Oncology Brain Metastases (RANO-BM) criteria strongly recommend the use of 1.5-mm or thinner MR images for detecting brain metastases [14]. However, the critical limitation is that there must be a trade-off between spatial resolution and noise [15]. Thin slices inherently lead to a decreased signal-to-noise ratio (SNR), which compromises image quality and may result in misdiagnosis.

In recent years, deep learning (DL)-based image enhancement has shown high potential in enhancing the image quality of thin-section MR imaging for small lesions, such as pituitary adenomas or multiple sclerosis plaques [15–17]. To our knowledge, no previous study has utilized DL image enhancement for thin-section BB MR imaging in patients with brain metastases. Therefore, we aimed to evaluate the utility of a commercially available vendor-neutral DL-based image enhancement for improving the image quality and diagnostic performance of 3D contrast-enhanced T1-weighted BB MR imaging for brain metastases.

## Materials and methods

This study was approved by the institutional review board of Seoul National University Hospital (IRB No. 2311-047-1482), and the requirement for informed consent was waived due to its retrospective nature.

### Patients

For the metastasis group, 181 consecutive patients with underlying malignancies who underwent a dedicated MRI protocol for the evaluation of brain metastasis between August 2023 and October 2023 were selected from our radiology report database. The inclusion criteria were as follows: patients who (1) had an underlying malignancy; (2) underwent MR imaging for the evaluation of brain metastasis (including both postcontrast BB and 3D gradient echo sequences) on 3-T scanners; and (3) had radiology reports indicating metastases in the brain parenchyma. The exclusion criteria were as follows: (1) suboptimal MR image quality; (2) MR images with more than 10 metastases; and (3) MR images with only completely cystic or hemorrhagic lesions.

For the non-metastasis group, 122 consecutive patients with underlying malignancies, who underwent the dedicated MRI protocol for the evaluation of brain metastasis between September 2023 and October 2023, were selected from our radiology report database. The inclusion criteria were as follows: patients (1) had underlying malignancy; (2) underwent MR imaging for the evaluation of brain metastasis (including both BB and 3D gradient echo sequences) on 3-T scanners; and (3) had radiology reports including “no evidence of metastasis” or its equivalents. The exclusion criterion was the same as criterion (1) for the metastasis group.

### MRI acquisition

MRI scans were performed with multiple 3-T scanners with a mixed variety of manufacturers (Discovery MR750w, GE Healthcare (*n* = 40); Signa Premier, GE Healthcare (*n* = 54); Ingenia 3.0 T CX, Philips Healthcare (*n* = 96); Biograph mMR, Siemens Healthineers (*n* = 18); Magnetom Skyra, Siemens Healthineers (*n* = 39)). The specific imaging parameters for each of the scanners are available in Supplementary Table 1. As a contrast agent, gadobutrol (Gadovist, Bayer; IV 0.1 mmol/kg) was administered. The dedicated MRI protocol for the evaluation of brain metastasis included pre- and postcontrast 3D T1-weighted gradient echo sequences and postcontrast 3D T1-weighted BB imaging.

### DL-based image enhancement

The postcontrast 3D axial BB images were post-processed with a commercially available DL-based software (SwiftMR v2.0.1.0, AIRS Medical Inc.). The underlying DL framework and its training process are summarized as follows.

SwiftMR is based on the well-known U-Net architecture [18]. The contracting path is a repetition of encoder blocks, consisting of two 3 × 3 convolutions with ReLU operations and a 2 × 2 max pooling step for downsampling. The expansive path uses successive decoder blocks to regain the spatial dimension while utilizing high-level features obtained from the contracting path and relatively detailed features from concatenation. Data consistency was enforced at each layer of the U-Net. Detailed information regarding this model can be found in [19]. The MR images used during the training and validation process of the model and those collected for this study were mutually exclusive.

### Quantitative image quality assessment

The SNR and contrast-to-noise ratio (CNR) were used for the quantitative assessment of the image quality of the metastasis group. For each standard or DL-enhanced BB image, a total of 3 ROIs were placed at the largest contrast-enhanced metastatic lesion, normal-appearing white matter (70 ± 9 mm^2^), and background (70 ± 9 mm^2^). The ROI of the metastatic lesion was set as the largest circle inside the enhanced region (25 ± 41 mm^2^). The SNRs of the metastases and the CNRs between the metastases and normal-appearing white matter were subsequently calculated using the following equations:

$${{\rm{SNR}}}=\frac{{{\rm{S}}}{{{\rm{I}}}}_{{{\rm{meta}}}}}{{{\rm{N}}}},{{\rm{CNR}}}=\frac{|{{\rm{S}}}{{{\rm{I}}}}_{{{\rm{meta}}}}-{{\rm{S}}}{{{\rm{I}}}}_{{{\rm{NAWM}}}}|}{{{\rm{N}}}}$$

where ${{\rm{S}}}{{{\rm{I}}}}_{{{\rm{meta}}}}$ and ${{\rm{S}}}{{{\rm{I}}}}_{{{\rm{NAWM}}}}$ refer to the mean SI of metastasis and normal-appearing white matter, respectively, and N refers to the standard deviation of background SI.

### Qualitative image quality assessment

Two neuroradiologists (S.W.J. and H.J.C., with 14 and 15 years of experience in radiology, respectively) independently assessed the image quality of the standard and DL-enhanced BB images in two reading sessions with at least a 2-week wash-out interval. Four hundred ninety-four axial BB images, consisting of 247 standard and 247 DL-enhanced MR images from 247 patients, were presented in a randomized crossover manner at each session to readers, who were blinded to the patient information. The readers evaluated the overall image quality and noise, using a 5-point Likert scale (1: not acceptable or no diagnostic value, 2: very limited diagnostic value, 3: acceptable for most diagnoses, 4: good for most diagnoses, and 5: optimal). The degree of artifacts was also evaluated using a 5-point Likert scale (1: unreadable (images of non-diagnostic quality), 2: severe artifact (images degraded but interpretable), 3: moderate artifact with some but no severe effect on diagnostic quality, 4: minimal artifact with no effect on diagnostic quality, and 5: no artifact).

### Diagnostic performance for brain metastases

The two readers also independently evaluated the lesion conspicuity using a 3-point scale (1: poor (barely seen), 2: fair, and 3: excellent). During the review, the readers were allowed access to corresponding postcontrast 3D axial gradient echo images for reference to decrease false positivity due to incomplete vessel suppression on postcontrast BB images.

For the reference standards, an experienced neuroradiologist (R.E.Y. with 15 years of experience in neuroradiology) retrospectively reviewed all MR images and annotated all the metastatic lesions. To improve the diagnostic accuracy for brain metastases, the radiologist referred to previous radiology reports during labeling.

### Statistical analysis

All statistical analyses were performed using R version 4.2.2 (The R Project for Statistical Computing). The quantitative and qualitative image qualities were compared between standard and DL-enhanced images using Wilcoxon signed-rank tests. Both lesion-based and patient-based approaches were used to evaluate the diagnostic performance of the readers. The sensitivity and conspicuity of the lesions were analyzed using logistic regression with generalized estimating equations (GEEs) and ordinal logistic regression with GEE, respectively. In the case of the patient-based approach, specificity was analyzed using logistic regression, while the average false-positive (FP) count was analyzed using Poisson regression. A *p*-value less than 0.05 was considered statistically significant.

## Results

### Baseline characteristics

**Fig. 1:**
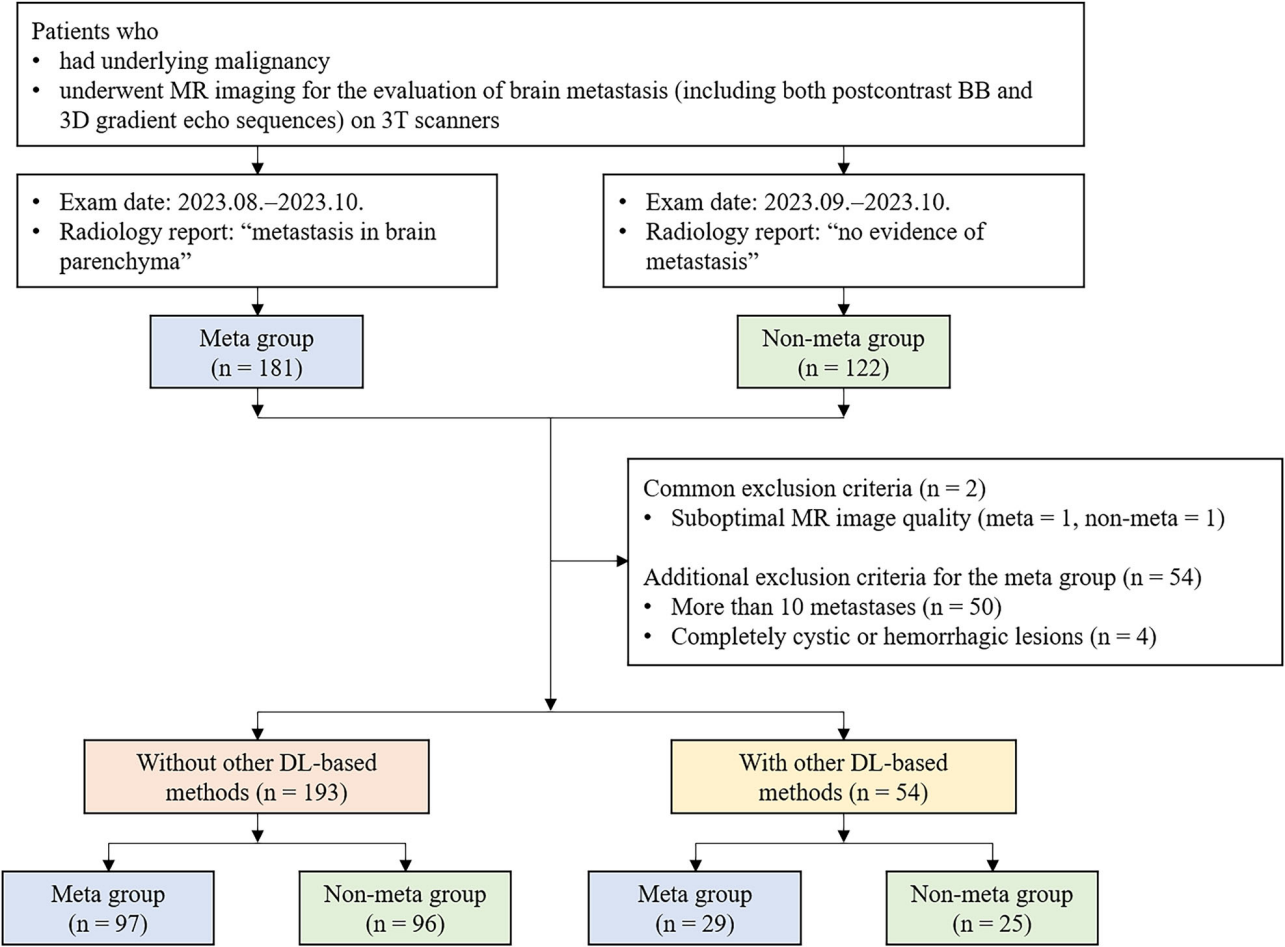
Flowchart for the study participant selection and image assessment. BB, black blood; Meta group, metastasis group; Non-meta group, non-metastasis group; DL, deep learning. ‘Without other DL-based methods’ refers to patients with MR images that were not previously processed using other DL-based methods. ‘With other DL-based methods’ refers to patients with MR images previously processed using AIR Recon DL^TM^, GE Healthcare

Of 181 consecutive patients with underlying malignancies in the metastasis group, 55 patients were excluded because their MR images had suboptimal image quality (*n* = 1), had more than 10 lesions (*n* = 50), or had completely cystic or hemorrhagic lesions (*n* = 4). Of 122 consecutive patients with underlying malignancies in the non-metastasis group, 1 patient was excluded because of suboptimal image quality. After exclusions, 126 patients with brain metastasis and 121 patients without metastasis were included (Fig. 1). Among these patients, 97 patients with metastasis (65 ± 12 years; 30 male, 67 female) and 96 patients without metastasis (65 ± 12 years; 51 male, 45 female) had MR images that were not previously processed using other DL-based methods (Table 1). The most common underlying malignancy was lung cancer. For the metastasis group, the mean lesion size was 7.6 mm (median, 5.4 mm (range, 1.6–58.9 mm)). The remaining 29 patients with metastasis (64 ± 10 years; 11 male, 18 female) and 25 patients without metastasis (66 ± 13 years; 16 male, 9 female) had MR images previously processed using another DL-based method (AIR Recon DL^TM^, GE Healthcare) (Supplementary Table 2).

**Table 1:** Baseline characteristics

Characteristics	Meta group (*n* = 97)	Non-meta group (*n* = 96)
Age (years)	65 ± 12	65 ± 12
Sex (female)	67 (69.1)	45 (44.8)
MRI scanner		
Discovery MR750w	24 (24.7)	16 (16.7)
Ingenia CX	49 (50.5)	47 (49.0)
Biograph mMR	9 (9.3)	9 (9.4)
Magnetom Skyra	15 (15.5)	24 (25.0)
Primary cancer		
Lung	70 (72.2)	74 (77.1)
Breast	15 (15.5)	7 (7.3)
Gastrointestinal^a^	0 (0.0)	4 (4.2)
Hepatobiliary^b^	0 (0.0)	0 (0.0)
Genitourinary/gynecologic^c^	7 (7.2)	5 (5.2)
Thyroid	2 (2.1)	3 (3.1)
Others^d^	3 (3.1)	3 (3.1)
Multiplicity		
1	34 (35.1)	-
2–3	35 (36.1)	-
4–10	28 (28.9)	-
Lesion size	7.59 ± 6.93	-

Age and lesion size are expressed as means ± standard deviations. Other variables are presented as frequencies (percentages)

*Meta group* metastasis group, *non-meta group* non-metastasis group

^a^ Gastrointestinal cancer includes gastric cancer and colorectal cancer

^b^ Hepatobiliary cancer includes hepatocellular carcinoma and cholangiocarcinoma

^c^ Genitourinary/gynecologic cancer includes renal cell carcinoma, bladder cancer, prostate cancer, testicular cancer, ovarian cancer, uterine cancer, and extragonadal germ cell tumor

^d^ Others include melanoma, lymphoma, olfactory neuroblastoma, liposarcoma, myoepithelial carcinoma, and metastasis of unknown origin

### Quantitative image quality assessment

Both the SNR and CNR were higher in the DL-enhanced images than in the standard images (*p* < 0.01 for both) (Table 2 and Supplementary Table 3). In the subgroup analysis according to the type of MRI scanner, both the SNR and CNR were higher in the DL-enhanced images than in the standard images across all subgroups, regardless of the scanner used (*p* < 0.01 for all) (Table 2).

**Table 2:** Quantitative image quality assessment

	Standard	DL-enhanced	*p*-value
SNR	438.3 (171.4, 1124.4)	661.1 (236.9, 1361.8)	< 0.01
Discovery MR750w (*n* = 24)	126.2 (103.8, 163.5)	144.1 (112.0, 197.4)	< 0.01
Ingenia CX (*n* = 49)	1124.4 (696.5, 1471.6)	1286.7 (901.7, 1857.6)	< 0.01
Biograph mMR (*n* = 9)	220.3 (157.8, 276.1)	293.7 (277.7, 661.1)	< 0.01
Magnetom Skyra (*n* = 15)	295.7 (239.5, 475.2)	409.4 (307.1, 692.6)	< 0.01
CNR	173.9 (66.5, 450.3)	223.5 (103.4, 566.0)	< 0.01
Discovery MR750w (*n* = 24)	63.7 (29.8, 93.8)	73.3 (39.2, 124.2)	< 0.01
Ingenia CX (*n* = 49)	393.7 (196.7, 758.8)	543.6 (296.5, 921.2)	< 0.01
Biograph mMR (*n* = 9)	52.6 (43.7, 105.4)	126.0 (103.4, 149.2)	< 0.01
Magnetom Skyra (*n* = 15)	122.1 (78.7, 255.3)	208.5 (114.7, 424.3)	< 0.01

Values are presented as medians (interquartile ranges)

*DL* deep learning, *SNR* signal-to-noise ratio, *CNR* contrast-to-noise ratio

### Qualitative image quality assessment

**Fig. 2:**
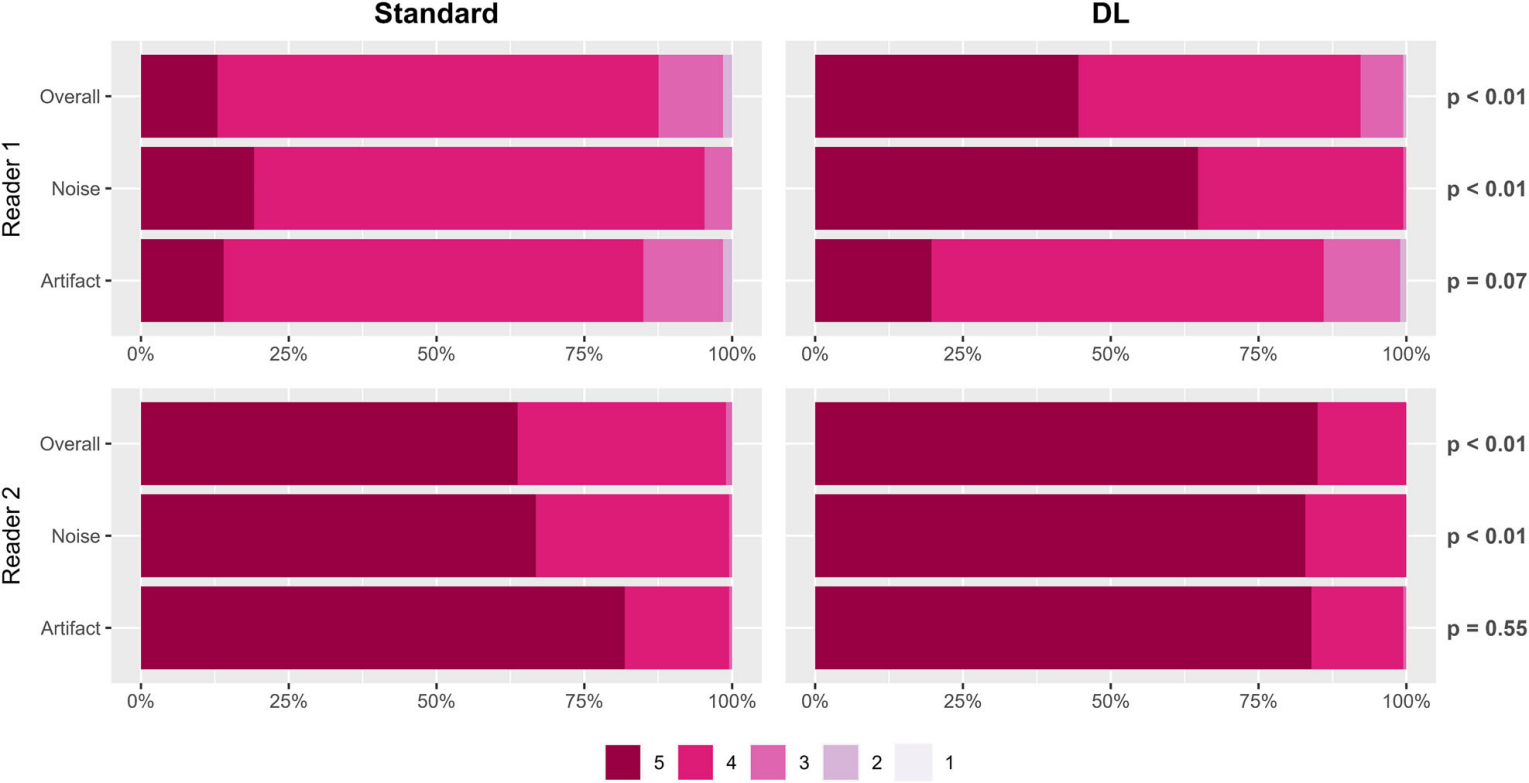
Qualitative image quality assessment. For the overall image quality and noise, 1: not acceptable or no diagnostic value, 2: very limited diagnostic value, 3: acceptable for most diagnoses, 4: good for the majority of diagnoses, and 5: optimal. For the degree of artifacts, 1: unreadable (images of non-diagnostic quality), 2: severe artifact (images degraded but interpretable), 3: moderate artifact with some but no severe effect on diagnostic quality, 4: minimal artifact with no effect on diagnostic quality, and 5: no artifact. DL, deep learning

The evaluations of the overall image quality, noise, and artifact are summarized in Fig. 2 and Supplementary Fig. 1. Both the overall image quality and noise were rated higher in the DL-enhanced images than in the standard images in both readers (*p* < 0.01 for all) for MR images that were not previously processed using other DL-based methods and in reader 1 (*p* = 0.04; *p* < 0.01) for those previously processed using AIR Recon DL^TM^. In contrast, the degree of artifacts did not significantly differ between the standard and DL-enhanced images for both readers (reader 1, *p* = 0.07 and *p* = 0.81; reader 2, *p* = 0.55 and *p* = 0.08).

### Diagnostic performance for brain metastases

**Fig. 3:**
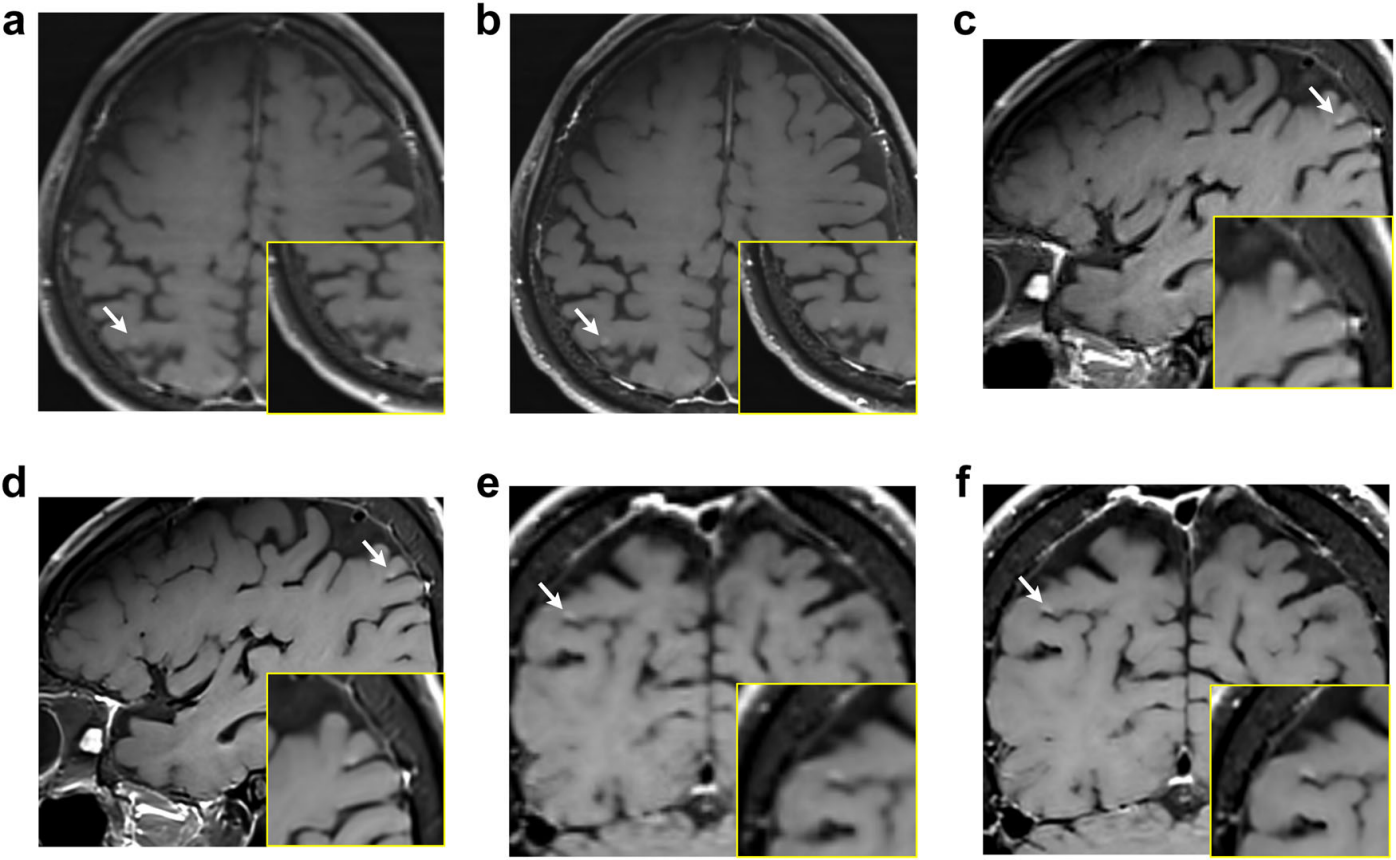
Case of a 67-year-old man with lung cancer. Standard (**a**, **c**, **e**) and deep learning (DL)-enhanced (**b**, **d**, **f**) contrast-enhanced T1-weighted black blood (BB) images. A tiny enhancing lesion at the right parietal cortex (arrow) is more conspicuous in the DL-enhanced image than in the standard image

The sensitivity per lesion in the standard and DL-enhanced images is presented in Table 3. The average reader sensitivity was significantly higher for the DL-enhanced images than for the standard images (86% vs. 79%, respectively; *p* = 0.02). In a subgroup analysis based on lesion size, metastases smaller than 5 mm were better detected in the DL-enhanced images than in the standard images (78% vs. 69%, respectively; *p* = 0.03), whereas the difference between the two images was not significant for larger lesions (5 mm ≤ size < 10 mm, *p* = 0.17; size ≥ 10 mm, *p* = 0.28). The representative case of a metastasis smaller than 5 mm is shown in Fig. 3.

**Table 3:** Sensitivity per lesion in standard and DL-enhanced images

	All metastases (*n* = 281)		Size < 5 mm (*n* = 123)		5 mm ≤ size < 10 mm (*n* = 96)		Size ≥ 10 mm (*n* = 62)	
	Sensitivity (%)	*p*-value	Sensitivity (%)	*p*-value	Sensitivity (%)	*p*-value	Sensitivity (%)	*p*-value
Reader 1								
Standard	87 (245)	Reference	81 (100)	Reference	92 (88)	Reference	92 (57)	Reference
DL	93 (260)	0.08	91 (112)	0.047*	94 (90)	0.60	94 (58)	0.73
Reader 2								
Standard	71 (198)	Reference	56 (69)	Reference	79 (76)	Reference	86 (53)	Reference
DL	79 (221)	0.07	65 (80)	0.20	88 (84)	0.17	92 (57)	0.28
Reader average								
Standard	79	Reference	69	Reference	85	Reference	89	Reference
DL	86	0.02*	78	0.03*	91	0.17	93	0.28

Values in brackets represent the actual number of detected lesions

*Size* size of metastatic lesion measured on a standard image, *DL* deep learning

* Statistically significant (*p* < 0.05)

The distribution of the lesion conspicuity scores is provided in Table 4. The lesion conspicuity scores differed between the standard and DL-enhanced images for both readers (*p* < 0.01 for both). After DL-based enhancement, the number of lesions that were unfound or received a conspicuity score of 1 decreased, whereas those that received a conspicuity score of 3 increased. Subgroup analysis according to lesion size also showed statistical differences in the distribution of the lesion conspicuity scores between the two images (reader 1: (size < 5 mm, *p* < 0.01), (size ≥ 10 mm, *p* = 0.02); reader 2: (size < 5 mm, *p* < 0.01), (5 mm ≤ size < 10 mm, *p* = 0.01), (size ≥ 10 mm, *p* = 0.01)), except for lesions sized equal to or larger than 5 mm but smaller than 10 mm in reader 1 (*p* = 0.06).

**Table 4:** Lesion conspicuity according to size

	No.	Standard No.				DL No.				*p*-value
		X	1	2	3	X	1	2	3	
Reader 1	281	36	24	70	151	21	21	68	171	< 0.01*
Size < 5 mm	123	23	17	35	48	11	15	38	59	< 0.01*
5 mm ≤ size < 10 mm	96	8	5	22	61	6	4	21	65	0.06
Size ≥ 10 mm	62	5	2	13	42	4	2	9	47	0.02*
Reader 2	281	83	3	19	176	60	2	25	194	< 0.01*
Size < 5 mm	123	54	1	10	58	43	1	12	67	< 0.01*
5 mm ≤ size < 10 mm	96	20	2	5	69	12	1	8	75	0.01*
Size ≥ 10 mm	62	9	0	4	49	5	0	5	52	0.01*

Data represent the number of lesions

*X* undetected lesion (i.e., lesion that did not receive a conspicuity score), *Size* size of metastatic lesion measured on a standard image, *DL* deep learning

* Statistically significant (*p* < 0.05)

**Fig. 4:**
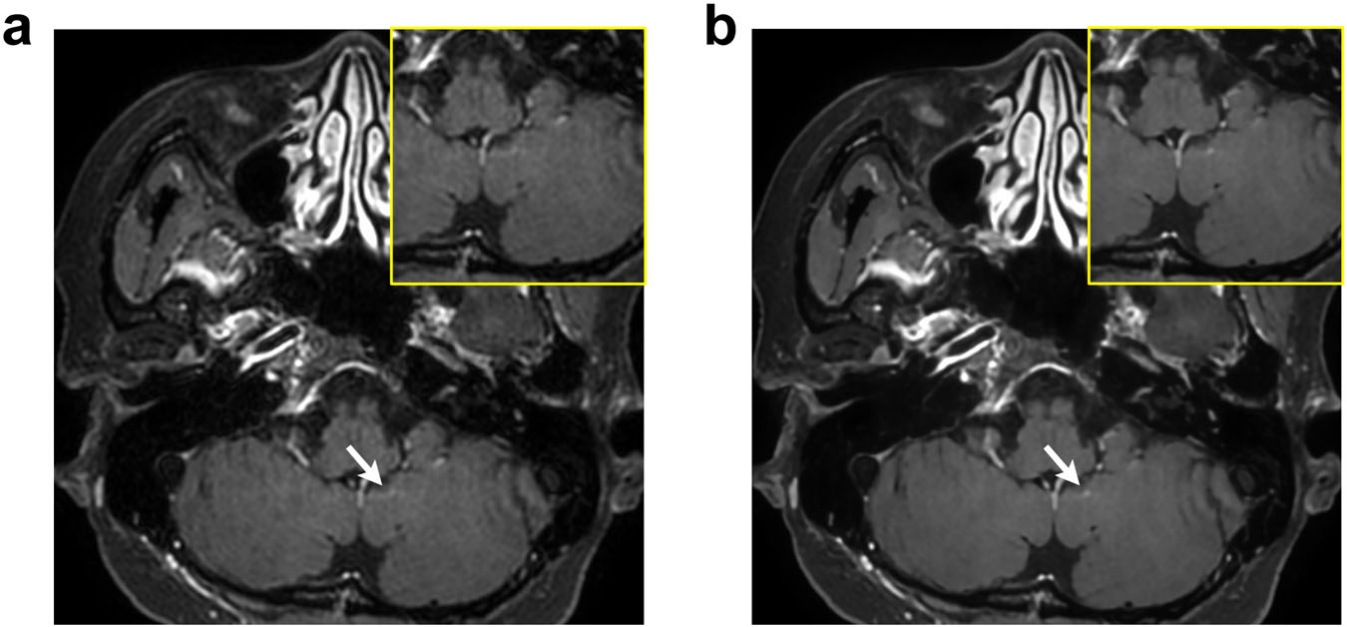
False-positive (FP) case of brain metastasis in a 43-year-old woman with breast cancer. Standard (**a**) and deep learning (DL)-enhanced (**b**) contrast-enhanced T1-weighted black blood (BB) images. A tiny dot-like enhancement due to an unsuppressed blood vessel at the left cerebellum (arrow) is more conspicuous in the DL-enhanced image than in the standard image

The specificity and average FP count per patient are shown in Table 5. The specificity per patient in the DL-enhanced images was comparable to that in the standard images (*p* = 0.24). The average FP count in the DL-enhanced images was also comparable to that in the standard images (*p* = 0.18). The representative FP case is presented in Fig. 4.

**Table 5:** Diagnostic performance per patient

	Specificity (%)	*p*-value	Average FP count	*p*-value
Reader 1				
Standard	94 (90/96)	Reference	0.13 (25/193)	Reference
DL	91 (87/96)	0.42	0.20 (38/193)	0.10
Reader 2				
Standard	99 (95/96)	Reference	0.04 (8/193)	Reference
DL	97 (93/96)	0.34	0.04 (7/193)	0.80
Reader average				
Standard	96	Reference	0.09	Reference
DL	94	0.24	0.12	0.18

Values in brackets represent the actual number of cases

*Average FP count* average number of false-positive lesions per patient, *DL* deep learning

Results for MR images previously processed using AIR Recon DL^TM^ are presented in Supplementary Tables 4–6. The number of lesions detected or receiving higher conspicuity scores tended to increase in the DL-enhanced images for lesions smaller than 5 mm, although the statistical significance was reached only for the distribution of conspicuity scores in reader 2 (*p* = 0.04). Specificity and average FP count in the DL-enhanced images were comparable to those of the standard images (*p* = 0.75; *p* = 0.52).

## Discussion

In this study, we evaluated the efficacy of DL-based image enhancement for the diagnosis of brain metastasis. This retrospective study compared standard BB brain images and their DL-enhanced versions in terms of quantitative and qualitative image quality and diagnostic performance for metastatic lesions. The SNR and CNR increased after DL enhancement regardless of vendor type. The overall image quality and noise level were preferable for the DL-enhanced images than for the standard images. As compared with the standard images, readers, on average, achieved higher lesion sensitivity with the DL-enhanced images. Changes in reader-perceived artifact, specificity, and average FP count did not reach statistical significance.

Brain metastases are 10 times more common compared to primary malignant brain tumors, making their detection important for radiologists [20]. However, accurate diagnosis can be difficult because metastases vary in size, shape, number, and location [21, 22]. According to Buller et al, the lesion sizes of untreated patients with multiple metastases tend to follow a power law distribution, usually with one large lesion and other smaller lesions [23]. Small lesions less frequently accompany diagnostic clues, such as edema, making the detection of small metastases more challenging compared to larger ones [24]. For these reasons, it is particularly notable that our study showed superior overall sensitivity on DL images than on standard images, especially for detecting small metastases (< 5 mm).

For the accurate diagnosis of small brain metastases, Li et al developed a two-stage DL detection and segmentation algorithm to reduce the FP rate and improve precision in diagnosing brain metastases on thin-section postcontrast T1-weighted images [25]. However, accurately diagnosing small metastases is still challenging due to the indistinguishable small vessels. For that reason, Oh et al used BB images to develop a DL-based detection algorithm for brain metastases [26]. However, with standard BB imaging, there are still limitations in making an accurate diagnosis of small metastases because they typically exhibit relatively weak lesion-to-background contrast, as compared with larger lesions. Our results have shown that the application of commercially available DL-based enhancement to BB images can reduce the false-negative rate by improving the SNR, CNR, and overall image quality.

Unlike other DL-based image enhancement software that is integrated directly into image acquisition pipelines, either in the raw data or k-space domains, the software used in this study operates in the Digital Imaging and Communications in Medicine (DICOM) domain in a post-processing manner [19]. The underlying DL model was trained using paired target and input DICOM images, which were conventionally reconstructed from k-space data and their multidimensionally degraded versions, respectively. Relationships between those paired images have been learned by the DL algorithm and are now utilized to reduce noise and enhance the resolution of unseen DICOM images from the real world. Along with image data, supplementary contextual data, including scan parameter information and the expected noise reduction factor, were implemented in the training process. Contextual data not only helped the model successfully learn the target-input relationships in diverse degradation settings but also enabled the tuning of noise reduction levels.

Anatomical structures are sharpened, and images are denoised through the image enhancement process. Previous studies obtained images with higher SNRs and CNRs by using the same software [27–31]. Yoo et al demonstrated an improved characterization of the cauda equina on DL-enhanced lumbar MR images, and Lee et al reported comparable subjective image quality of DL-enhanced knee MR images, while reducing scan times by an average of 32.3% and 41.0%, respectively [27, 28]. Others reported a better depiction of hippocampal lesions for diagnosing temporal lobe epilepsy or higher vessel conspicuity for MR angiography and intracranial vessel wall imaging [29–31]. While the former two studies focused on enhancing accelerated images to reduce scan time, we used the software to generate DL-enhanced images that surpassed standard images in terms of image quality and diagnostic accuracy, as in the latter three studies.

However, it is worth mentioning that existing artifacts and unsuppressed blood vessel signals may be emphasized during the same process. Several studies have reported a higher risk of FPs on BB than on conventional gradient echo imaging, due to incomplete vessel suppression [7, 32]. As the SNR and CNR improve, the number of cases where vessels are mistaken for metastases may increase after applying DL. In fact, the majority of FP cases in this study were due to misinterpretation of residual vessel signals. Nonetheless, the specificity, average FP count, and artifact scores of the DL images were all comparable to those of the standard images.

Our study has several limitations. First, this study was retrospectively conducted at a single center, and a prospective multicenter study is recommended to validate generalizability. Second, our study used a single commercially available DL-based MR image enhancement software to enhance BB MR images. Future work comparing SwiftMR with other commercially available DL-based MR image enhancement software is warranted to validate its clinical usefulness. Third, the number of enrolled patients was not sufficiently large. The relatively small sample size may have influenced some analyses, especially those of subgroups whose results did not reach statistical significance. Fourth, certain metastasis cases were excluded. Despite the non-negligible incidence of completely cystic or hemorrhagic metastases, they were excluded because of the difficulty in defining enhancing portions. In addition, patients with more than 10 lesions were excluded, as defining each lesion would be too arduous in such instances. Fifth, the reference standards for the diagnosis of brain metastases were established based on radiologic findings, mostly without pathologic confirmation. Many patients with brain metastasis do not typically undergo surgical resection, and thus, a highly experienced neuroradiologist thoroughly reviewed all available data to establish alternative criteria.

In conclusion, DL-based enhancement improves the image quality and diagnostic performance of 3D contrast-enhanced T1-weighted BB MR imaging for the detection of small brain metastases. This could be a great support to neuroradiologists, as metastatic brain tumors have a wide variety of sizes, shapes, numbers, and locations, leading to difficulties in diagnosis. Further large-scale prospective studies are needed to validate the value of DL-enhanced BB images in clinical practice.

## Supplementary information

ELECTRONIC SUPPLEMENTARY MATERIAL

## Abbreviations

BB: Black blood
CNR: Contrast-to-noise ratio
DL: Deep learning
FP: False-positive
GEE: Generalized estimating equation
SNR: Signal-to-noise ratio
