# Correction: Deep learning-based image enhancement for improved black blood imaging in brain metastasis

**DOI:** 10.1007/s00330-026-12348-3

**Published:** 2026-02-24

**Authors:** Gaeun Oh, Seungyoon Paik, Sang Won Jo, Hye Jeong Choi, Roh-Eul Yoo, Seung Hong Choi

**Affiliations:** 1 Seoul National University College of Medicine, Seoul, Republic of Korea; 2 Department of Radiology, Dongtan Sacred Heart Hospital, Hallym University College of Medicine, Hwaseong-si, Republic of Korea; 3 Department of Radiology, CHA University Bundang Medical Center, Seongnam-si, Republic of Korea; 4 Department of Radiology, Seoul National University Hospital, Seoul, Republic of Korea; 5 Center for Nanoparticle Research, Institute for Basic Science (IBS), Seoul, Republic of Korea; 6 School of Chemical and Biological Engineering, Seoul National University, Seoul, Republic of Korea

**Correction to: European Radiology**

10.1007/s00330-025-11920-7published online 08 August 2025

In the original article, the affiliation of author Sang Won Jo was incorrectly given as “Department of Radiology, Kangdong Sacred Heart Hospital, Seoul, Republic of Korea”. The correct affiliation is “Department of Radiology, Dongtan Sacred Heart Hospital, Hallym University College of Medicine, Hwaseong-si, Gyeonggi-do, Republic of Korea”.

The original article has been corrected.

