# Supplementary material for: Deep learning-based image enhancement for improved black blood imaging in brain metastasis
Source: Eur Radiol. 2025 Aug 8;36(2):928–38. doi: 10.1007/s00330-025-11920-7 (PMC12953279; doi:10.1007/s00330-025-11920-7)
Supplement: Supplementary file 1 — ELECTRONIC SUPPLEMENTARY MATERIAL [file 330_2025_11920_MOESM1_ESM.pdf]

Deep learning-based image enhancement for improved black blood imaging in brain metastasis

ELECTRONIC SUPPLEMENTARY MATERIAL

Supplementary Table 1: MRI scan parameters

	**Discovery MR750w**	**Signa Premier**	**Ingenia CX 3.0T**	**Biograph mMR**	**Magnetom Skyra**
Field strength (T)	3.0	3.0	3.0	3.0	3.0
Head coil channel	32	48	32	32	64
**3D contrast-enhanced T1-weighted BB**					
TR (ms)	350	550	500	700	700
TE (ms)	27.0	21.0	29.6	12.0	11.0
FA (°)	90.0	90	90.0	120.0	120.0
NEX	1	1	1	1	1
Matrix	256 × 256	256 × 256	252 × 252	320 × 271	256 × 228
Section thickness (mm)	1	1	1	1	1
Intersection gap (mm)	0	0	0	0	0
FOV (mm)	256 × 256	256 × 256	250 × 250	230 × 256	228 × 256
**3D T1-weighted gradient echo**					
TR (ms)	2560	2310	8.5	1700	2020
TE (ms)	2.8	2.0	4.6	3.1	4.0
FA (°)	8.0	10.0	8.0	9.0	12.0
NEX	1	1	1	1	1
Matrix	256 × 256	256 × 256	256 × 256	256 × 256	256 × 256
Section thickness (mm)	1	1	1	1	1
Intersection gap (mm)	0	0	0	0	0
FOV (mm)	256 × 256	256 × 256	250 × 250	250 × 250	250 × 250

BB, black blood; TR, repetition time; TE, echo time; FA, flip angle; NEX, number of excitations; FOV, field of view.

Eur Radiol (2025) Oh G, Paik S, Jo SW, Choi HJ, Yoo RE, Choi SH.

Supplementary Table 2: Baseline Characteristics (Signa Premier)

Characteristics	Meta group (n = 29)	Non-meta group (n= 25)
Age (years)	64 ± 10	66 ± 13
Sex (female)	18 (62.1)	9 (36.0)
Primary cancer		
Lung	15 (51.7)	16 (64.0)
Breast	7 (24.1)	1 (4.0)
Gastrointestinal[a]	1 (3.4)	2 (8.0)
Hepatobiliary[b]	0 (0.0)	4 (16.0)
Genitourinary/Gynecologic[c]	3 (10.3)	1 (4.0)
Thyroid	2 (6.9)	0 (0.0)
Others[d]	1 (3.4)	1 (4.0)
Multiplicity		
1	10 (34.5)	-
2-3	10 (34.5)	-
4-10	9 (31.0)	-
Lesion size	10.17 ± 9.71	-

Age and lesion size are expressed as means ± standard deviations. Other variables are presented as frequencies (percentages).

[a] Gastrointestinal cancer include gastric cancer and colorectal cancer.

[b] Hepatobiliary cancer include hepatocellular carcinoma and cholangiocarcinoma.

[c] Genitourinary/Gynecologic cancer include renal cell carcinoma, bladder cancer, prostate cancer, testicular cancer, ovarian cancer, uterine cancer, and extragonadal germ cell tumor.

[d] Others include melanoma, lymphoma, olfactory neuroblastoma, liposarcoma, myoepithelial carcinoma, and metastasis of unknown origin.

Meta group = metastasis group, Non-meta group = non-metastasis group.

Supplementary Table 3: Quantitative image quality assessment (Signa Premier)

	Standard	DL-enhanced	*p* value
SNR (n = 29)	438.6 (288.5, 613.6)	716.7 (490.4, 1022.7)	< 0.01
CNR (n = 29)	170.7 (100.2, 289.6)	316.2 (180.7, 421.5)	< 0.01

Values are presented as medians (interquartile ranges). DL = deep learning, SNR = signal-to-noise ratio, CNR = contrast-to-noise ratio.

Supplementary Table 4: Sensitivity per lesion in standard and DL-enhanced images (Signa Premier)

	All metastases [n = 85]		Size < 5mm [n = 32]		5mm ≤ Size < 10mm [n = 24]		Size ≥ 10mm [n = 29]	
	**Sensitivity (%)**	***p* value**	**Sensitivity (%)**	***p* value**	**Sensitivity (%)**	***p* value**	**Sensitivity (%)**	***p* value**
Reader 1								
Standard	93 [79]	Reference	84 [27]	Reference	96 [23]	Reference	100 [29]	Reference
DL	95 [81]	0.46	91 [29]	0.43	96 [23]	> 0.99	100 [29]	> 0.99
Reader 2								
Standard	85 [72]	Reference	72 [23]	Reference	88 [21]	Reference	97 [28]	Reference
DL	86 [73]	0.79	81 [26]	0.39	83 [20]	0.66	93 [27]	0.48
Reader average								
Standard	89	Reference	78	Reference	92	Reference	98	Reference
DL	91	0.55	86	0.22	90	0.72	97	0.53

Values in brackets represent the actual number of detected lesions. Size = size of metastatic lesion measured on a standard image, DL = deep learning.

Supplementary Table 5: Lesion conspicuity according to size (Signa Premier)

	No.	Standard No.				DL No.				*p* value
		X	1	2	3	X	1	2	3	
Reader 1	85	6	7	23	49	4	7	21	53	0.29
Size < 5mm	32	5	7	11	9	3	7	8	14	0.13
5mm ≤ Size < 10mm	24	1	0	9	14	1	0	9	14	> 0.99
Size ≥ 10mm	29	0	0	3	26	0	0	4	25	-[a]
Reader 2	85	13	0	5	67	12	0	4	69	0.49
Size < 5mm	32	9	0	5	18	6	0	3	23	0.04*
5mm ≤ Size < 10mm	24	3	0	0	21	4	0	0	20	-[a]
Size ≥ 10mm	29	1	0	0	28	2	0	1	26	-[a]

Data represent the number of lesions. X = undetected lesion, i.e. lesion that did not receive a conspicuity score, Size = size of metastatic lesion measured on a standard image, DL = deep learning, * = statistically significant ($p < 0.05$).

[a] Number of conspicuity score categories with at least one corresponding lesion is less than three.

Supplementary Table 6: Diagnostic performance per patient (Signa Premier)

	Specificity (%)	*p* value	Average FP count	*p* value
Reader 1				
Standard	92 [23/25]	Reference	0.30 [16/54]	Reference
DL	96 [24/25]	0.56	0.28 [15/54]	0.86
Reader 2				
Standard	84 [21/25]	Reference	0.22 [12/54]	Reference
DL	84 [21/25]	> 0.99	0.33 [18/54]	0.28
Reader average				
Standard	88	Reference	0.26	Reference
DL	90	0.75	0.31	0.52

Values in brackets represent the actual number of cases. Average FP count = average number of false-positive lesions per patient, DL = deep learning.

Supplementary Figure 1: Qualitative image quality assessment (Signa Premier)

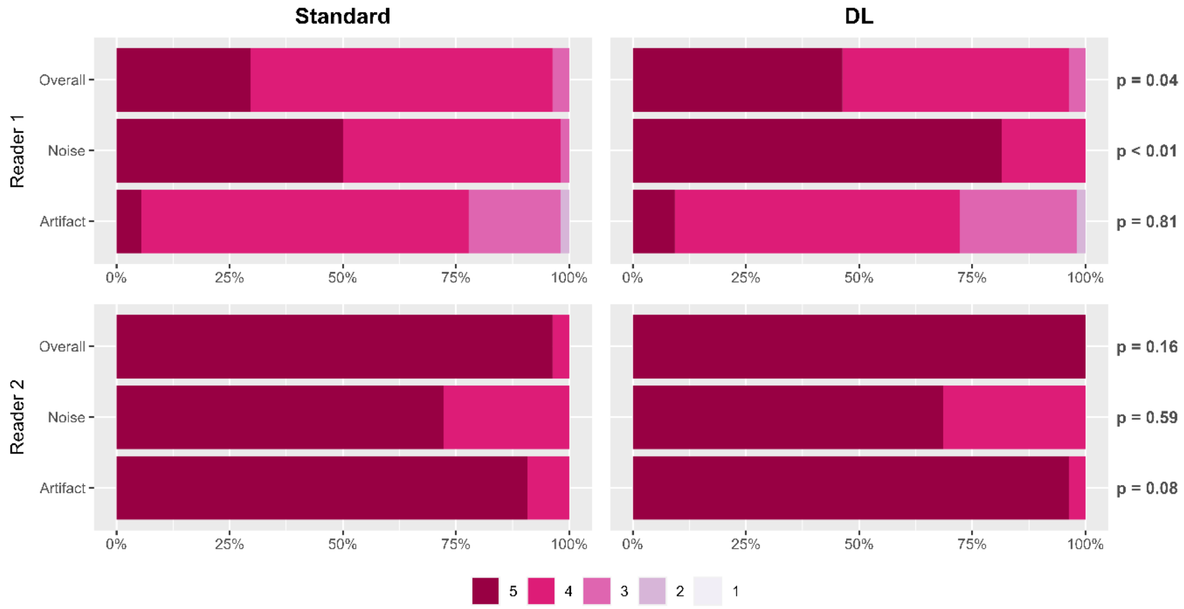

For the overall image quality and noise, 1: not acceptable or no diagnostic value, 2: very limited diagnostic value, 3: acceptable for most diagnoses, 4: good for the majority of diagnoses, and 5: optimal. For the degree of artifacts, 1: unreadable [images of non-diagnostic quality], 2: severe artifact [images degraded but interpretable], 3: moderate artifact with some but no severe effect on diagnostic quality, 4: minimal artifact with no effect on diagnostic quality, and 5: no artifact. DL = deep learning.
